# Repeated in-field radiosurgery for locally recurrent brain metastases: Feasibility, results and survival in a heavily treated patient cohort

**DOI:** 10.1371/journal.pone.0198692

**Published:** 2018-06-06

**Authors:** Panagiotis Balermpas, Susanne Stera, Jens Müller von der Grün, Britta Loutfi-Krauss, Marie-Thérèse Forster, Marlies Wagner, Christian Keller, Claus Rödel, Volker Seifert, Oliver Blanck, Robert Wolff

**Affiliations:** 1 Department of Radiation Oncology, University Hospital Johann Wolfgang Goethe University, Frankfurt, Germany; 2 Saphir Radiosurgery Center, Frankfurt, Germany; 3 German Cancer Research Center (DKFZ), Heidelberg, Germany; 4 German Cancer Consortium (DKTK) partner site: Frankfurt am Main, Germany; 5 Department of Neurosurgery, University Hospital Johann Wolfgang Goethe University, Frankfurt, Germany; 6 Institute for Neuroradiology, University Hospital Johann Wolfgang Goethe University, Frankfurt, Germany; 7 Department of Radiation Oncology, University Medical Center Schleswig-Holstein, Kiel, Germany; George Washington University, UNITED STATES

## Abstract

**Purpose:**

Stereotactic radiosurgery (SRS) is an established primary treatment for newly diagnosed brain metastases with high local control rates. However, data about local re-irradiation in case of local failure after SRS (re-SRS) are rare. We evaluated the feasibility, efficacy and patient selection characteristics in treating locally recurrent metastases with a second course of SRS.

**Methods:**

We retrospectively evaluated patients with brain metastases treated with re-SRS for local tumor progression between 2011 and 2017. Patient and treatment characteristics as well as rates of tumor control, survival and toxicity were analyzed.

**Results:**

Overall, 32 locally recurrent brain metastases in 31 patients were irradiated with re-SRS. Median age at re-SRS was 64.9 years. The primary histology was breast cancer and non-small-cellular lung cancer (NSCLC) in respectively 10 cases (31.3%), in 5 cases malignant melanoma (15.6%). In the first SRS-course 19 metastases (59.4%) and in the re-SRS-course 29 metastases (90.6%) were treated with CyberKnife^®^ and the others with Gamma Knife. Median planning target volume (PTV) for re-SRS was 2.5 cm^3^ (range, 0.1–37.5 cm^3^) and median dose prescribed to the PTV was 19 Gy (range, 12–28 Gy) in 1–5 fractions to the median 69% isodose (range, 53–80%). The 1-year overall survival rate was 61.7% and the 1-year local control rate was 79.5%. The overall rate of radiological radio-necrosis was 16.1% and four patients (12.9%) experienced grade ≥ 3 toxicities.

**Conclusions:**

A second course of SRS for locally recurrent brain metastases after prior local SRS appears to be feasible with acceptable toxicity and can be considered as salvage treatment option for selected patients with high performance status. Furthermore, this is the first study utilizing robotic radiosurgery for this indication, as an additional option for frameless fractionated treatment.

## Introduction

Brain metastases are diagnosed in up to 40% of patients with solid primary tumors outside the central nervous system and the incidence is continuously increasing [1]. Stereotactic radiosurgery (SRS) is an established primary treatment for newly diagnosed, untreated brain metastases as well as for resection cavities after previous neurosurgical operation, with high level of evidence for both indications [2–5]. More important, stand-alone SRS is considered as standard-of-care treatment method, especially when patients have a limited number of lesions (usually 1–4) and is represented in the most national recommendations and guidelines [6, 7]. SRS provides excellent 1-year-local control rates (LCR) of 65%-90% regardless of histology, as has been already shown in many prospective and retrospective studies [2, 3, 8–10]. Unfortunately, the median survival in all of these series amounted to merely 8–11 months.

However, novel systemic agents like targeted therapies and immune check point inhibitors have recently improved survival of metastatic patients [11–15], so that the relative rare event of a local recurrence of a metastasis previously treated with SRS could become increasingly important. In such cases surgery [16, 17] or whole-brain radiotherapy (WBRT) [18, 19] are currently the most common treatment practices, although both of these treatment modalities present some problems. Surgery is often not possible, otherwise the patient would have been resected in the first-line, or even in the case of resection it has been shown that this option alone does not provide sufficient control rates [3, 5, 20]. On the other hand, WBRT leads to a relative quick and frequent decline in cognitive function [4, 21] and it would be desirable to avoid or defer this treatment if not absolutely necessary, especially for this often extracranially controlled patient subgroup with a limited number of metastases.

Despite the fact that data examining the feasibility end efficacy of repeated SRS for newly developed distant to previously treated brain metastases already exist, few reports exist about a repeated SRS for the same, recurrent lesion (re-SRS), i.e. a second course of SRS in the same place (“in loco”) [22]. Most of the published studies consist of very small cohorts and either heterogeneous groups of local and distant recurrences mixed with heterogeneous initial treatments with only very few patients really receiving two courses of SRS for the same metastasis [22, 23]. Reasons for this lack of data could be mainly attributed to the assumption of increased toxicity after applying a high-dose re-irradiation. Moreover, only in recent years a sufficient number of patients experienced a clinically relevant local failure due to improvements in systemic disease control. The aim of this study was therefore to retrospectively evaluate our experience regarding feasibility, efficacy and patient selection characteristics in treating locally recurrent brain metastases with a second course of radiosurgery in SRS-dedicated platforms.

## Patients and methods

### Data acquisition

For this retrospective analysis, we searched the internal database of our radiosurgery center, after institutional review board approval (ethical committee of the Frankfurt university, number: 401/17). All patient data were fully anonymized and the ethics committee waived the requirement for informed consent for this retrospective trial. In order to identify all of the patients with brain metastases treated more than once, independent of the exact localization. The records, including all neuro-radiological reports and the corresponding images and plans of these patients were then reviewed independently by two of the treating physicians (P.B. and R.W.) and only cases with unambiguous anatomic overlap of at least one of the treated gross tumor volumes (GTV) in two different radiosurgery series were included for further evaluation. This means that an intersection of the two GTVs was necessary for a case in order to be included in this analysis and had to be verified by both reviewers. No one of the such identified cases has been excluded for other reasons. The same medical records were used for assessment of patient characteristics and oncological endpoints and technical characteristics of each treatment were acquired directly from the planning systems.

### SRS treatment and follow-up

For all patients, contrast enhanced T1 weighted MRI of the brain with 1 mm slice thickness, reconstructed in all 3 dimensions, was used for primary delineation of the gross target volume (GTV) and organs at risks. The planning target volume (PTV) was defined as the GTV without any further margin. A stereotactic frame (Leksell G Frame^®^, Elekta) was used for immobilization for patients treated with the Gamma Knife System (Leksell Gamma Knife 4C^®^). For CyberKnife (Accuray, Sunnyvale, USA) SRS thermoplastic masks were used for patient immobilization and patient localization during treatment was done with stereoscopic x-ray image guidance. A 1 mm thin slice planning CT with according MRI image registration was accomplished. Treatment planning was performed using MultiPlan (Accuray) for the CyberKnife and Leksell Gamma Plan^®^ 8.3.1 (Elekta) for the Gamma Knife System according to international best practice guidelines [24, 25]. SRS was mostly applied in a single fraction with the exceptions of cases with a GTV diameter > 3 cm where a three-to-five fraction regimen was used.

**Fig 1:**
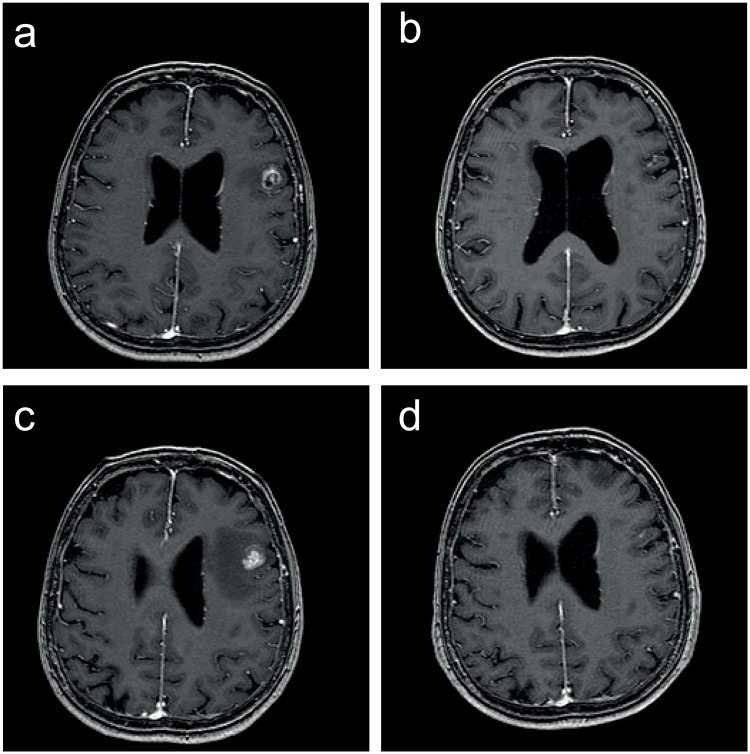
MRI images of a recurrent metastasis. a) before and b) after initial treatment, c) before and d) after Re-SRS.

Patient follow-up consisted of serial MRI-scans (same as for treatment planning) every 8–12 weeks after SRS. Multiple MRIs over a short period of time (if needed every 6–8 weeks) were conducted in order to diagnose tumor-growth. A continuous increase in the size of a lesion (defined as area of contrast enhancement) and contrast uptake in at least two sequential MRI series, non-responsive to steroids, combined with corresponding perfusion-weighted data was defined as local recurrence. For differentiation between tumor progression and pseudo-progression all clinical and radiological data were used. Radiological data in patients with brain metastases routinely included unenhanced T2-, FLAIR-, T1- susceptibility- and diffusion-weighted images, ADC-maps, contrast-enhanced T1-weighted images and perfusion-weighted images, especially CBV-maps. In selected cases with inconclusive routine imaging 1H spectroscopic chemical shift imaging was added [26–30]. Since 2016, all neuro-oncology patients treated with immunotherapy in our institution are evaluated according to the iRANO criteria for tumor response [31]. The perfusion-weighted, contrast enhanced MRI, including the additional images as described above, was the main method for differentiation between progression and pseudo-progression/ necrosis. No biopsies or PET-scans were used for definition of recurrence in this patient cohort. All cases and treatment decisions were discussed in a multidisciplinary tumor board in the presence of a specialized neuroradiologist, neurosurgeon, neurooncologist and radiation oncologist. An example of a recurrent metastasis at time of initial SRS and before and after re-SRS and the corresponding treatment plan is depicted as Fig 1.

### Patients

A total of 31 patients were treated for 32 recurrent brain metastases between 06/2011 and 06/2017. First diagnosis of the primary malignancy was between 06/1996 and 09/2015, i.e. a median of 42.6 months (range, 11.8–208.6 months) before re-irradiation and the first occurrence of brain metastasis between 11/2004 and 07/2016, with a median of 16.5 months (range, 4–110.6 months) until re-SRS. Of all patients, 16 (51.6%) were female and 15 were male (48.4%). The median age of the patients at the time of the first SRS was 64 years and at the time of re-SRS 65 years (range, 43–81 years).

### Statistical analysis

Follow-up intervals were defined from the date of SRS (first course of SRS or re-SRS, respectively) to the date of the respective event or last contact. Local recurrence (as defined above) was counted as an event for the endpoint local control rate (LCR). Radiological progression of the primary tumor or already described metastases, as well as emergence of new metastases and local recurrence or death were counted as events for disease free survival (PFS). Finally, death from any cause was counted as an event for the endpoint overall survival (OS). The Kaplan-Meier method and Log-rank test were used for univariate analysis for all time-to event endpoints and the Fisher’s exact test for the analysis of categorical data. Cox-regression and a backwards selection method were used for multivariate analyses. P-values ≤ 0.05 were considered significant. All statistics were performed using IBM-SPSS version 21 (IBM Corp, Armonk, NY, USA).

## Results

### Patient and tumor characteristics

Of all patients treated, 77.4% initially presented with a Karnofsky Performance Score (KPS) of 90–100%. This percentage was somewhat reduced in the re-SRS treatment to 61.3%, but no patients with KPS < 60% were treated. Primary histology was breast cancer (BC) and non-small-cell lung cancer (NSCLC) each in 10 cases (31.3%), whereas 5 patients suffered from metastases of malignant melanoma (15.6%). The remaining 7 metastases were of different histology. 7 of the breast cancer patients were Her-2-positive (3 of them also hormone receptor positive), one patient triple-negative and two patients were hormone-receptor positive, Her-2-negative. Regarding the NSCLC patients, 6 of them presented with adenocarcinoma (only one with an EGFR mutation and no one with ALK/ROS mutations) and 4 of them with squamous cell carcinoma. Nine (28.1%) re-treated brain metastases were each located in the frontal lobe or the cerebellum, 7 (21.9%) metastases were in the parietal lobe and further 7 in other brain regions. An overview of patient and tumor characteristics can be found in Table 1 and for the cases treated with single-fraction Re-SRS also separately in S1 Table.

**Table 1:** Patient and tumor characteristics.

		Total	%
**Patients**		31	
**Lesions**		32	
**Gender**	Male	15	48.4
	Female	16	51.6
**Age 1**^**st**^ **SRS**	Median (range) in years	64.0	(41.0–80.4)
**Age Re-SRS**	Median (range) in years	64.86	(42.6–81.3)
**Karnofsky-Index at 1**^**st**^ **SRS**	Median (range) in %	90	(60–100)
**Karnofsky-Index at Re-SRS**	Median (range) in %	90	(60–100)
**Primary tumor**			
**(per lesion)**	NSCLC	10	31.3
	Melanoma	5	15.6
	Breast cancer	10	31.3
	Renal cell cancer	1	3.1
	Colorectal cancer	1	3.1
	Other	5	15.6
**Localization of recurrent lesions**			
	Frontal	9	28.1
	Temporal	2	6.3
	Parietal	7	21.9
	Occipital	1	3.1
	Thalamus/mesencephalon	3	9.4
	Cerebellum	9	28.1
	Brainstem	1	3.1
**Time intervals between SRS-series**	Median (range) in months	12.4	(3.2–88.2)

Abbreviations: SRS: stereotactic radiosurgery, NSCLC: non-small cellular lung cancer.

### Treatment characteristics

Dedicated radiosurgery platforms (Gamma Knife and CyberKnife) were used for all treatments. Specifically, 19 of the metastases (59.4%) in the first SRS-course and 29 of the metastases (90.6%) in the re-SRS-course were treated with the CyberKnife. The median number of metastases per patient treated in both series was 1, but the range varied: up to 10 metastases per patient in the initial treatment and up to 3 metastases per patient in the second course. Although single fraction radiotherapy was applied in the vast majority of cases (93.8% in the first series and 75% in the second), 3 to 5 fraction regimens were also used. The most common fractionation prescribed for the non-single fraction cases was 3x 8Gy, also 5x 5 Gy and 4x 6 Gy were used on the discretion of the treating physician. Median cumulative PTV was 2.9 cm^3^ (range: 0.2–22.9 cm^3^) and 2.8 cm^3^ (range: 0.1–37.5 cm^3^) and the median PTV per lesion was 2.0 cm^3^ (range: 0.1–22.9 cm^3^) and 2.5 cm^3^ (range: 0.1–37.5 cm^3^) for initial and re-SRS, respectively. The median prescription isodose was 65% for the first SRS and 69% for the re-SRS and the median dose prescribed on this encompassing isodose was 18 Gy (median BED_10_ 50.4 Gy) and 19 Gy (median BED_10_ 50.4 Gy), respectively. The median PTV mean and the median PTV maximum doses for the first treatment were 23.8 Gy (median BED_10_mean: 79.3 Gy) and 29.5 Gy (median BED_10_max: 110 Gy) and for the second treatment were 23.5 Gy (median BED_10_mean: 70.6 Gy) and 28.0 Gy (median BED_10_max: 90.2 Gy), respectively. The median time interval between the two SRS treatments was 12.4 months (range, 3.2–88.2 months). A complete/ near complete remission following initial therapy was observed in 12 (37.5%) of the cases. In all the other cases the tumor regressed but a residual lesion could be observed. In all of these 20 cases (62.5%) where a recurrence was diagnosed and treated after incomplete regression, an increase in size and contrast uptake in at least 2 consecutive MRIs associated with an increase in perfusion (rCBV and rCBF values) could be observed. For completely asymptomatic patients these MRIs were carried out in an interval of 10–12 weeks and for symptomatic patients sometimes in a shorter interval of 6–8 weeks under steroid medication. No tumor that did not fulfill these criteria and showed only persistence without increasing in size, contrast-enhancement and perfusion was treated.

Various other local and systemic therapies were also implemented before, after and re-SRS. Specifically, either chemotherapy (incl. anti-hormone treatment) or targeted therapy were applied concomitant to re-SRS in a total of 14 patients (43.8%). More precisely, one of the patients with NSCLC received anti-EGFR-targeted therapy concomitant to re-SRS and regarding the patients with breast cancer, all of the 5 patients with hormone-dependent tumors had an ongoing anti-hormone treatment and 3 out of 7 Her-2-positive patients an ongoing anti-Her-2-therapy. Furthermore, 10 of the patients have received immunotherapy at some point in their medical history, but in only 5 of the 32 cases (15.6%) this treatment was applied simultaneously or within 3 months of the re-SRS. 5 patients (16.1%) also received whole brain radiotherapy (WBRT), 4 of them before and 1 of them after the two SRS-series (none in between) and the median number of total brain irradiation series for all patients was 3 (range, 2–7, including SRS for other localizations). Furthermore, 12 patients (38.7%) had at least one surgical resection of brain metastases in their history, 9 of them (29%) including the target lesion. Only one of these cases concerns an incomplete resection in the interval between the two SRS courses. In the other cases the surgical treatment was performed either at diagnosis (6 patients) or due to a later progression/symptom aggravation (2 patients, one with a “real” progression and one with symptomatic necrosis). An overview of treatment characteristics is depicted in Tables 2–4.

**Table 2:** Treatment characteristics: 1^st^ SRS.

		Total	%
**Number of Lesions treated at 1**^**st**^ **SRS**			
	Median (range)	1	(1–10)
	1	21	65.6
	2	7	21.9
	3	1	3.1
	6	1	3.1
	7	1	3.1
	10	1	3.1
**Number of fractions 1**^**st**^ **SRS**			
	1	30	93.8
	3	2	6.2
**Platform 1**^**st**^ **SRS**			
	CyberKnife	19	59.4
	Gamma Knife	13	40.6
**Cumulative PTV 1**^**st**^ **SRS**	Median (range) in cm^3^	2.9	(0.22–22.88)
**PTV 1**^**st**^ **SRS**	Median (range) in cm^3^	2.0	(0.1–22.9)
**Enclosing isodose 1**^**st**^ **SRS**	Median % (range)	65	(32–78)
**Prescribed dose 1**^**st**^ **SRS** **Dose BED**_**10**_	Median (range) in Gy	18 50.4	(15–24) (37.5–70.4)
**Mean PTV dose 1**^**st**^ **SRS** **Dose BED**_**10**_	Median (range) in Gy	23.8 79.3	(18.0–31.1) (50.5–113.9)
**Maximum dose 1**^**st**^ **SRS** **Dose BED**_**10**_	Median (range) in Gy	29.5 110	(22.1–44.0) (70.7–237.6)

Abbreviations: SRS: stereotactic radiosurgery, PTV: planned target volume, BED: biological equivalent dose, Gy: Gray.

**Table 3:** Treatment characteristics: Re-SRS.

		Total	%
**Number of Lesions treated by Re-SRS**			
	Median (range)	1	(1–3)
	1	24	75.0
	2	5	15.6
	3	3	9.4
**Number of fractions Re-SRS**			
	1	24	75.0
	3	6	18.6
	4	1	3.2
	5	1	3.2
**Platform Re-SRS**			
	CyberKnife	29	90.6
	Gamma Knife	3	9.4
**Cumulative PTV Re-SRS**	Median (range) in cm^3^	2.8	(0.1–37.5)
**PTV Re-SRS**	Median (range) in cm^3^	2.5	(0.1–37.5)
**Enclosing isodose Re-SRS**	Median % (range)	69	(53–80)
**Prescribed dose Re-SRS** **Dose BED**_**10**_	Median (range) in Gy	19 50.4	(12–28) (26.4–60)
**Mean PTV dose Re-SRS** **Dose BED**_**10**_	Median (range) in Gy	23.5 70.6	(14.3–33.0) (34.5–89.9)
**Maximum dose Re-SRS** **Dose BED**_**10**_	Median (range) in Gy	28.0 97.2	(17.4–38.1) (40.1–126.3)
**Systemic therapy during Re-SRS**		14	43.8

Abbreviations: SRS: stereotactic radiosurgery, PTV: planned target volume, BED: biological equivalent dose Gy: Gray.

**Table 4:** Treatment characteristics: Other treatments.

		Total	%
**Whole brain radiotherapy**			
	Total	5	16.1
	Before SRS	4	12.5
	After two SRS-series	1	3.1
**Surgery**			
	Total	12	38.7
	Including target lesion	9	28.1
	Target lesion only	5	15.6
	Target lesion and other	4	12.5
	Other lesion	3	9.4
**Number of brain irradiation series**	Median (range)	3	(2–7)
	2	13	40.6
	3	12	37.5
	4	4	12.5
	6	2	6.3
	7	1	3.1

Abbreviations: SRS: stereotactic radiosurgery, BED: biological equivalent dose, Gy: Gray.

### Tumor control, survival and toxicity

**Fig 2:**
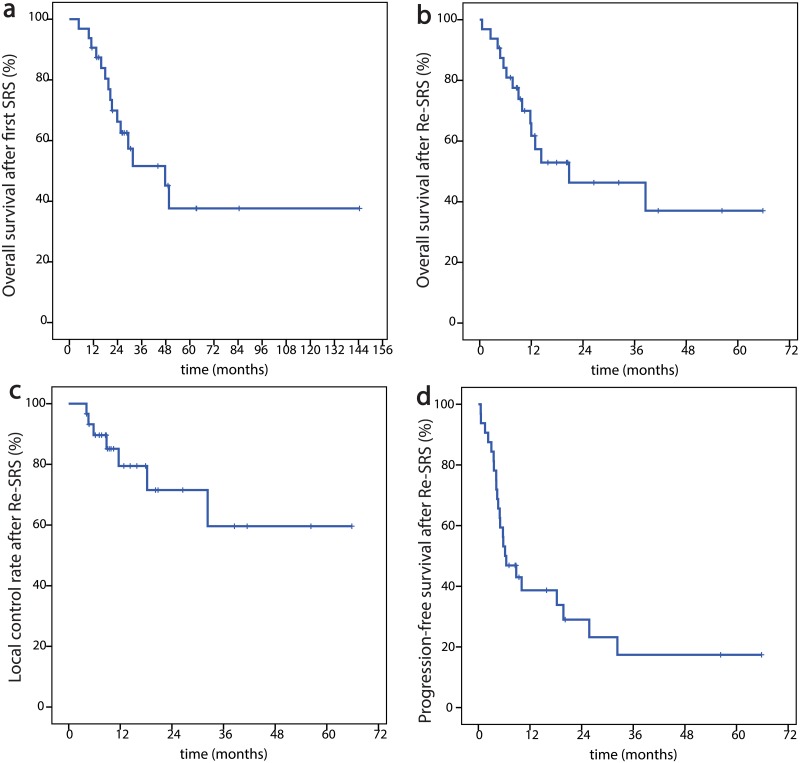
Kaplan-Meier curves of oncological endpoints. **a)** overall survival after first SRS, **b)** overall survival after Re-SRS, **c)** local control rate after Re-SRS, **d)** progression-free survival after Re-SRS.

After a median follow up of 11.9 months (range, 0.6–65.8 months) the 1-year-OS was 61.7% and the 2-year-OS 46.3%. Regarding the PFS we found the 1-year-PFS to be 38.7% and 2-y-PFS to be 29.0%. Finally, the LCR was 79.5% after one year and 71.5% after two years Fig 2.

**Fig 3:**
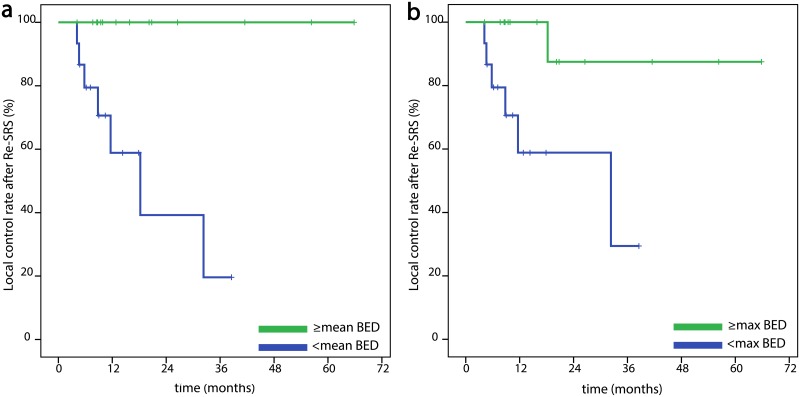
Kaplan-Meier curves of local control rate after Re-SRS. **a)** dependent on BEDmean (≥ median vs. lower) **b)** dependent on BEDmax (≥ median vs. lower).

Despite the small number of patients and events we also conducted exploratory univariate and multivariate analyses for the three oncological endpoints (OS, PFS, LCR) above. We included following factors with possible impact on these endpoints in univariate analyses: sex, age (≥ median vs. younger), histology (breast cancer vs. other, NSCLC vs. other and finally radioresistant histologies: melanoma-renal cell carcinoma-colorectal cancer vs. other), general condition during re-SRS as KPS (90–100% vs. lower and 100% vs. lower), number of metastases irradiated in the second SRS course (re-irradiated lesion alone vs. more lesions), exact localization of the target lesion (e.g. cerebellum vs. other and frontal lobe vs. other and thalamus-brainstem-cerebellum vs. other), PTV of the re-irradiated lesion (≥ median vs. smaller), maximum re-SRS dose (BED_max_ ≥ median vs. lower), PTV mean re-SRS dose (BED_mean_ ≥ median vs. lower), prescription isodose (≥ median vs. lower), number of fractions (1 vs. more), systemic treatment during re-SRS, surgical excision in the previous history and finally time interval between the two radiotherapy courses of the target lesion (≥ median vs. shorter) Fig 3. Regarding OS, female sex significantly correlated with a longer survival and the same was true for breast cancer histology, a prescription isodose lower than 69% (median), neurosurgical excision in the previous history and longer time interval between the two SRS series (p = .036, p = .035, p = 0.04, p = .009 and p = .012, respectively). None of these factors remained significant in multivariate analysis. Equally, female sex, breast cancer histology, longer time interval and lower than median prescription isodose significantly correlated with a longer PFS (p = .019, p = .001, p = .011 and p = .009, respectively). Moreover, higher BED_max_ (p = .011) and higher PTV BED_mean_ (p = .013) had a significantly positive influence on PFS. In the multivariate analysis BED_max_ (p = .021) and breast cancer histology (p = .001) were significantly associated with better PFS. Both a higher BED_max_ and a higher BED_mean_ showed a significant association with better local control (p = .013 and p = .002, respectively) in univariate, but not in multivariate analysis and interestingly, breast cancer (vs. rest) (p = .971) or “radio-resistant” histology as defined above (melanoma, renal and colorectal cancer) (p = .609) showed no correlation with local control rate. However, these results are biased by the very low number of events for this endpoint (7 local recurrences) and the statistically significant correlation between BED_mean/encompassing_ ≥ median and a target volume smaller than median (p = .032/p = .002 respectively) and therefore need careful interpretation. The two hormone-positive and Her-2-negative breast cancer patients, receiving concomitant anti-hormone treatment, as well as the one NSCLC patient with EGFR-mutation, receiving anti-EGFR-targeted therapy, were still locally controlled at the last follow-up and all of them already show longer local control and survival than the median.

A separate analysis, including only cases treated with single-fraction Re-SRS, was also conducted, the results are depicted in S2 and S3 Tables and S1 Fig.

### Toxicity

Out of 31 patients 25 (80.6%) experienced no toxicity after the re-SRS treatment. On the other hand, 5 patients had radiological signs of necrosis, with or without clinical correlate (16.1%). Of these, 4 patients (12.9%) experienced grade III-IV toxicities which may be attributed to the radiotherapy, namely one case of bleeding with hemiparesis, two cases of frequent seizures (one of them with preexisting seizures) and one case of symptomatic necrosis, with symptoms which resolved after surgery. In some of these cases the differentiation between SRS-toxicity and tumor progression was not easy. The two remaining patients only showed radiological signs of necrosis. No significant correlation between dose (either physical, nor BED), prescription isodose, time interval between SRS series or PTV could be found. The localization of the Re-SRS did also seem not to play a role for adverse events, even when we divided the cases in “sensible” localization (brainstem-thalamus-cerebellum”) versus the rest (p = .625 for necrosis and p = .776 for grade III-IV toxicities). An overview of the results regarding oncological endpoints and toxicity can be found in Table 5. Interestingly, there was no case with grade III-IV toxicity in the patients treated with single-fraction Re-SRS (S3 Table). Of the 5 patients with necrosis only one had received previous whole brain radiotherapy. Of the two patients experiencing seizures, one was on antiepileptic medication (carbamazepine) due to a previous event and the symptoms aggravated after Re-SRS and one experienced new seizures without having any previous events or medication. Both of them had a metastasis that involved –at least partly- the temporal lobe. We could not find any anticoagulation in the files of the patient experiencing the symptomatic bleeding and no other factors which possibly correlated with the above side effects. The only patient receiving anti-EGFR therapy (for NSCLC) did not show any signs of severe toxicity or necrosis. Of the patients receiving anti-Her-2-treatment (7 patients with breast cancer), only one developed a radiological necrosis (without clinical symptoms). Regarding immunotherapy, of the 10 patients receiving immunotherapy at some point in their medical history only one developed severe toxicity (NSCLC patient with necrosis and seizures), but the immunotherapy was started only several months after these symptoms occurred, due to further systemic progression. A second patient with receiving immunotherapy for renal cell carcinoma concomitant to the re-SRS developed a radiological necrosis, without any clinical symptoms.

**Table 5:** Oncological endpoints and toxicities.

		%	No. of patients
**Overall survival**			
	1 year	61.7	
	2 years	46.3	
**Progression-free survival**			
	1 year	38.7	
	2 years	29.0	
**Local control rate**			
	1 year	79.5	
	2 years	71.5	
**Toxicity**			
	Any grade	19.4	6
	Grade III-IV	12.9	4
	Radiological signs of necrosis	16.1	5

## Discussion

The optimal treatment for recurrent brain metastases after initial therapy remains unclear and until now no high-level evidence for definitive recommendations exists. Since the number of patients with brain metastases is continuously increasing and because of more efficient systemic therapies and early diagnosis leading to prolonged life expectancy [32], the need for salvage options for those experiencing local recurrences after initial stereotactic radiosurgery (SRS) is more demanding than ever. Whole-brain radiotherapy (WBRT) and surgery are common practices for these situations, but both of them have significant disadvantages and limitations. Surgery remains an effective salvage treatment option, but its invasive character and its potential risks for these palliative care patients may limit surgical indication. Thus, many brain metastases are unresectable, most already before the initial SRS treatment. Furthermore, surgery does not provide very high local control rates as stand-alone treatment [3, 5, 20], with recurrence rates at the original site between 22% and 48% [16, 33, 34]. WBRT, on the other hand, increases the rate of later neurocognitive deterioration [4, 21] and compromises patients’ quality of life [35], especially for long-term survivors with only a few metastases, so that its use for a limited number of metastases in patients with longer life expectancy cannot be justified. Furthermore, an in-field-recurrence after initial high dose SRS must generally be considered as a relatively radio-resistant lesion, so that the commonly applied WBRT-doses are not expected to offer long-term tumor control. The results after chemotherapy for this indication are even worse, with local control of 2–4 months and survival of 3–7 months [36–38].

It has been shown that repeated courses of stereotactic radiosurgery can successfully be used for treating new brain metastases after initial SRS in order to defer WBRT [39, 40]. In a retrospective analysis of 95 patients with 652 metastases, the median OS after the second course of SRS was 11 months and adverse events were observed in only 2% of the cases treated [39]. These encouraging results and the lack of effective alternatives raise the obvious question if a repeated, “in loco”, second course of SRS could also be used for treating locally recurrent brain metastases in a pre-irradiated region.

Only four recently published, but relatively small and retrospective cohorts have addressed this question so far and the radiotherapy methods were very heterogeneous among each other. In the oldest and largest study published, Minniti et al. reported on 43 patients treated with 3 fractions of 7–8 Gy by using a gantry-based linear accelerator resulting in 1-year OS of 37% and 1-year-LCR of 70% [41]. Notably, no single-fraction SRS was used. The overall rate of radio-necrosis was 19% and of symptomatic necrosis 14%. Importantly, the local control rate was significantly higher for breast cancer histology and the Karnofsky performance status significantly affected the survival rate. In the only other published gantry-based linear accelerator series of re-SRS, Rana et al. re-treated 28 patients, 59% of them with single-fraction regimen and achieved 1-year OS of 90.6% and 1-year-LCR of 88.3% [42]. The overall rate of radio-necrosis was 18.8% and occurred only in lesions treated with single-fraction SRS. The other two studies retrospectively examining re-SRS both used the Gamma Knife system [43, 44]. Cohorts in these studies consisted of 32 and 22 patients, respectively, having been treated in a single fraction in all cases. However, the median prescribed re-SRS dose in the first study was 20.0 Gy, whereas in the second one only 15.5 Gy. This could possibly partially explain the slightly different results regarding 1-year-LCR: 79.0% and 61.1% respectively (OS was 70% and 37.5%, respectively). Nevertheless, those first series utilizing higher doses, also reported of 24% symptomatic radiation necrosis. This rate was much lower in the series of Koffer et al., prescribing 15.5 Gy and observing radiation necrosis in only 9.2% of the cases. In both of the studies the necrosis rate was significantly associated with the volume irradiated, however, a difference was that in the first publication the cumulative dose applied to the respective volume (> 40 Gy) also was a significant factor for necrosis. All of the authors above, regardless of the platform used, conclude that repeated SRS as salvage therapy is feasible with acceptable toxicities. Intriguingly, there was no case with grade III-IV toxicity in the patients treated with single-fraction Re-SRS in our analysis. Although the number of patients is too low to draw safe conclusions, we noted this finding and think that it could be associated with selection bias: patients treated with more fractions were the ones presented with larger recurrences (>3 cm diameter) as already stated in the methods section. Moreover, an extrapolation of the observations made in this study to postoperative (“adjuvant”) situations after previous SRS, where larger target volumes are a common issue, is not possible, as the target lesion was surgically treated before the re-SRS only in one of the present cases and even in this case the second radiosurgery was targeted at a macroscopic residual tumor. As the re-SRS was a postoperative SRS only in this one case no statistical analysis for this special constellation was possible.

The present series, reporting 31 patients, is among the largest so far. From the studies using a single-fraction regimen, only McKay et al. treated one more patient and moreover, to the best of our knowledge, this study is presenting the first results of re-SRS with robotic radiosurgery (CyberKnife^®^) more easily allowing for a fractionated SRS if the PTV becomes too large. The local control rate after one year in our series was 79.5% and the OS 61.7%, hence comparable to the other published data. In the 5 cases with simultaneous immunotherapy included here, there was no ambiguity regarding response, because the first response evaluation has always taken place after a –sufficient- 3 months-period [31] and the patients also had sequential MRI imaging after that, in which no delayed response could be observed. Similar results, meanwhile confirmed from different authors and using different radiotherapy platforms, show that a second course of SRS for local recurrences after initial SRS is a reasonable option, and that control rates achieved are –if at all- only slightly lower than those achieved by first-line treatment [45] [46]. Regarding toxicity, the rate of radio-necrosis in our series did not exceed the rates observed in the literature (see above) and lies somewhere between the rates observed in the two series which used only single-fraction treatment. The rate of serious adverse events of 12.9% (4 cases) is comparably high, but in 3 of these 4 cases the retrospective differentiation between radiation toxicity and tumor progression remained very difficult even after histologic evaluation in one case. However, the rate of severe toxicities of up to 9–20%, also reported in the other studies, should motivate to careful patient selection and evaluation of all alternative treatment options before prescribing a re-SRS.

It should be noted that uni- and multivariate analyses conducted in this study are only exploratory and therefore need careful interpretation, due to the limited numbers of events and the large number of factors analyzed. However, comparing present results with those of the other 4 studies described above common features can be identified, possibly facilitating future patient selection for such an approach. Thus, breast cancer histology was a significant positive predictor (at least univariate) for OS/PFS and one of the most common primary histology of the patients who were re-treated. Furthermore, the median time-interval between both SRS series was over 12 months and patients with an interval longer than this showed a significantly improved PFS. In the publication of Rana et al., the median interval between the two SRS courses was 10.7 months and in that of McKay et al.19.0 months. Hence, intervals in all these reports were clearly longer than both the median time to local failure (6.6 months) and median time to death (10.0 months) reported in the largest individual patient data meta-analysis of SRS [47].

Finally, almost all of the patients of the present study presented in excellent or good general condition (as expressed by means of KPS). This indicates that patients with a good performance status, an already prolonged survival and oligo-metastatic disease (as more commonly seen in breast cancer) may benefit most from a re-SRS approach for local recurrences. Additionally, and according to the present results, a higher maximum dose (and hence lower prescription isodose), positively influences local control and patient survival. Although a dose-response-relationship is known for SRS of brain metastases [48], these data are not mature enough to suggest a specific minimum inhomogeneity for re-SRS, which however may be needed, if the local recurrent metastasis is in fact deemed radio-resistant by definition of failure of initial SRS. On the other hand, some authors [42, 43] found a significant correlation of prescribed dose or prescription isodose with radio-necrosis, so that further dose escalation (compared to the initial SRS) should be practiced very carefully, although such a relationship could not be identified in the present data. Data about a direct correlation of mean or maximum biologically effective dose (BED) and outcome, especially in the reirradiation setting are rare, however some authors could show a general correlation of higher doses and better local control after primary, first-line, SRS for brain metastases [49, 50]. Here, the BED also significantly correlated with the volume of the target lesion (PTV), a bias attributed to the retrospective nature of the study and the tendency of the treating physicians to prescribe lower cumulative doses to larger volumes, so no safe conclusions about a control-dose correlation can be drawn.

This is the first study implementing robotic radiosurgery (CyberKnife^®^) for a second course of SRS for local recurrences after initial SRS as an additional technique, providing the option for frameless, fractionated treatment. Nevertheless, this study is aimed at showing proof of concept for the role of re-SRS in treating locally recurrent metastases and is not a study proving any superiority of a platform against another. On the contrary, the Gamma Knife was also used here and the results presented are comparable to that of other authors [23, 27, 28, 34], using other methods.

### Limitations of this study

The main limitations of this study, as of the other studies previously reported, are the limited number of patients and events and its retrospective design and finally the heterogeneous cohort and treatment characteristics. However, this series reporting on 32 metastases is one of the largest so far.

## Conclusion

The present results support that a second course of SRS for locally recurrent brain metastases after prior local SRS is feasible for selected patients with acceptable, but far from negligible toxicity. Therefore, re-SRS can be considered as salvage treatment option for patients with high performance status, similarly to the cohort presented here, after careful evaluation of possible serious adverse events. Further validation in a larger multi-center database or in prospective clinical trials is highly warranted.

## Supporting information

S1 Fig Oncological outcomes after single fraction SRS. a) overall survival after single fraction Re-SRS, b) local control rate after single fraction Re-SRS, c) progression-free survival after single fraction Re-SRS.
(EPS)

S1 Table Patient and tumor characteristics for cases treated with single-fraction Re-SRS. Abbreviations: SRS: stereotactic radiosurgery, NSCLC: non-small cellular lung cancer.
(DOCX)

S2 Table Treatment characteristics for cases treated with single-fraction Re-SRS. Abbreviations: SRS: stereotactic radiosurgery, PTV: planning target volume, Gy: Gray.
(DOCX)

S3 Table Oncological endpoints and toxicities for cases treated with single-fraction Re-SRS. (DOCX)
