# Supplementary material for: Repeated in-field radiosurgery for locally recurrent brain metastases: Feasibility, results and survival in a heavily treated patient cohort
Source: PLoS One. 2018 Jun 6;13(6):e0198692. doi: 10.1371/journal.pone.0198692 (PMC5991396; doi:10.1371/journal.pone.0198692)
Supplement: S1 Table — Abbreviations: SRS: stereotactic radiosurgery, NSCLC: non-small cellular lung cancer. (DOCX) [file pone.0198692.s002.docx]

Supplementary Table 1. Patient and tumor characteristics for cases with single-fraction Re-SRS

|  |  |  |  | **Total** | **%** |
| --- | --- | --- | --- | --- | --- |
| **Patients** |  |  |  | 23 |  |
| **Lesions** |  |  |  | 24 |  |
|  |  |  |  |  |  |
| **Gender** |  | Male |  | 10 | 43.5 |
|  |  | Female |  | 13 | 56.5 |
|  |  |  |  |  |  |
| **Age 1^st^ SRS** |  | Median (range) in years |  | 64.3 | (41.8-80.4) |
| **Age Re-SRS** |  | Median (range) in years |  | 65.0 | (42.8-81.3) |
| **Karnofsky-Index 1^st^ SRS** |  | Median (range) in % |  | 90 | (60-100) |
| **Karnofsky-Index Re-SRS** |  | Median (range) in % |  | 90 | (60-100) |
|  |  |  |  |  |  |
| **Primary tumor** |  |  |  |  |  |
| **(per lesion)** |  | NSCLC |  | 7 | 29.2 |
|  |  | Melanoma |  | 5 | 20.8 |
|  |  | Breast cancer |  | 9 | 37.5 |
|  |  | Colorectal cancer |  | 1 | 4.2 |
|  |  | Other |  | 2 | 8.3 |
| **Localization of recurrent lesions** |  |  |  |  |  |
|  |  | Frontal |  | 8 | 33.3 |
|  |  | Temporal |  | 1 | 4.2 |
|  |  | Parietal |  | 5 | 20.8 |
|  |  | Thalamus/mesencephalon |  | 2 | 8.3 |
|  |  | Cerebellum |  | 7 | 29.2 |
|  |  | Brainstem |  | 1 | 4.2 |
|  |  |  |  |  |  |
| **Time intervals between SRS-series** |  | Median (range) in months |  | 12.7 | (4.1-88.2) |

*Abbreviations: SRS: stereotactic radiosurgery, NSCLC: non-small cellular lung cancer*
