# Supplementary material for: Repeated in-field radiosurgery for locally recurrent brain metastases: Feasibility, results and survival in a heavily treated patient cohort
Source: PLoS One. 2018 Jun 6;13(6):e0198692. doi: 10.1371/journal.pone.0198692 (PMC5991396; doi:10.1371/journal.pone.0198692)
Supplement: S2 Table — Abbreviations: SRS: stereotactic radiosurgery, PTV: planning target volume, Gy: Gray. (DOCX) [file pone.0198692.s003.docx]

Supplementary Table 2. Treatment characteristics for cases with single-fraction Re-SRS

|  |  |  |  | **Total** | **%** |
| --- | --- | --- | --- | --- | --- |
| **Number of Lesions treated 1^st^ SRS** |  |  |  |  |  |
|  |  | Median (range) |  | 1 | (1-10) |
|  |  | 1 |  | 15 | 62.5 |
|  |  | 2 |  | 5 | 20.8 |
|  |  | 3 |  | 1 | 4.2 |
|  |  | 6 |  | 1 | 4.2 |
|  |  | 7 |  | 1 | 4.2 |
|  |  | 10 |  | 1 | 4.2 |
| **Number of Lesions treated Re-SRS** |  |  |  |  |  |
|  |  | Median (range) |  | 1 | (1-3) |
|  |  | 1 |  | 17 | 70.8 |
|  |  | 2 |  | 4 | 16.7 |
|  |  | 3 |  | 3 | 12.5 |
| **Number of fractions 1^st^ SRS** |  |  |  |  |  |
|  |  | 1 |  | 23 | 95.8 |
|  |  | 3 |  | 1 | 4.2 |
| **Number of fractions Re-SRS** |  |  |  |  |  |
|  |  | 1 |  | 24 | 100 |
| **Platform 1^st^ SRS** |  |  |  |  |  |
|  |  | CyberKnife |  | 14 | 58.3 |
|  |  | Gamma Knife |  | 10 | 417 |
| **Platform Re-SRS** |  |  |  |  |  |
|  |  | CyberKnife |  | 21 | 87.5 |
|  |  | Gamma Knife |  | 3 | 12.5 |
|  |  |  |  |  |  |
| **Cumulative PTV 1^st^ SRS** |  | Median (range) in cm^3^ |  | 2.2 | (0.2-22.9) |
| **PTV 1^st^ SRS** |  | Median (range) in cm^3^ |  | 1.2 | (0.1-22.9) |
| **Cumulative PTV Re-SRS** |  | Median (range) in cm^3^ |  | 2.3 | (0.1-14.9) |
| **PTV Re-SRS** |  | Median (range) in cm^3^ |  | 2.1 | (0.1-14.9) |
| **Enclosing isodose 1^st^ SRS** |  | Median % (range) |  | 64.5 | (32.0-75.0) |
| **Prescribed dose 1^st^ SRS** |  | Median (range) in Gy |  | 18.0 | (16.0-24.0) |
| **Mean PTV dose 1^st^ SRS** |  | Median (range) in Gy |  | 24.0 | (13.0-31.1) |
| **Maximum dose 1^st^ SRS** |  | Median (range) in Gy |  | 29.5 | (24.7-50.0) |
| **Enclosing isodose Re-SRS** |  | Median % (range) |  | 69.0 | (53.0-80.0) |
| **Prescribed dose Re-SRS** |  | Median (range) in Gy |  | 18.0 | (12.0-20.0) |
| **Mean PTV dose Re-SRS** |  | Median (range) in Gy |  | 23.3 | (14.3-25.4) |
| **Maximum dose Re-SRS** |  | Median (range) in Gy |  | 27.3 | (17.4-30.9) |
|  |  |  |  |  |  |
| **Systemic therapy during Re-SRS** |  |  |  | 12 | 50.0 |
| **Whole brain radiotherapy** |  |  |  |  |  |
|  |  | Total |  | 2 | 8.3 |
|  |  | Before SRS |  | 2 | 8.3 |
|  |  | After two SRS-series |  | 0 | 0 |
| **surgery** |  |  |  |  |  |
|  |  | Total |  | 9 | 37.5 |
|  |  | Including target lesion |  | 3 | 12.5 |
|  |  | Target lesion only |  | 3 | 12.5 |
|  |  | Target lesion and other |  | 3 | 12.5 |
|  |  | Other lesion |  | 0 | 0 |
|  |  |  |  |  |  |
| **Number of brain irradiation series** |  | Median (range) |  | 3 | (2-7) |
|  |  | 2 |  | 9 | 37.5 |
|  |  | 3 |  | 8 | 33.3 |
|  |  | 4 |  | 4 | 16.7 |
|  |  | 6 |  | 2 | 8.3 |
|  |  | 7 |  | 1 | 4.2 |

*Abbreviations: SRS: stereotactic radiosurgery, PTV: planning target volume, Gy: Gray*
