# Supplementary material for: Repeated in-field radiosurgery for locally recurrent brain metastases: Feasibility, results and survival in a heavily treated patient cohort
Source: PLoS One. 2018 Jun 6;13(6):e0198692. doi: 10.1371/journal.pone.0198692 (PMC5991396; doi:10.1371/journal.pone.0198692)
Supplement: S3 Table — (DOCX) [file pone.0198692.s004.docx]

Supplementary Table 3. Oncological endpoints and toxicities for cases with single-fraction Re-SRS

|  |  | **%** | **patients** |
| --- | --- | --- | --- |
| **Overall survival after Re-SRS** |  |  |  |
|  | 1 year | 63.8 |  |
|  | 2 years | 54.6 |  |
|  |  |  |  |
| **Progression-free survival** |  |  |  |
|  | 1 year | 44.4 |  |
|  | 2 years | 38.1 |  |
|  |  |  |  |
| **Local control rate** |  |  |  |
|  | 1 year | 74.4 |  |
|  | 2 years | 59.5 |  |
|  |  |  |  |
| **Toxicity** |  |  |  |
|  | Any grade | 8.3 | 2 |
|  | Grade III-IV | 0 | 0 |
|  | Radiological signs of necrosis | 8.3 | 2 |

*Abbreviation: SRS: stereotactic radiosurgery*
